# Neonatal cholestasis is an early liver manifestation of children with acid sphingomyelinase deficiency

**DOI:** 10.1186/s12876-022-02310-0

**Published:** 2022-05-09

**Authors:** Neng-Li Wang, Jing Lin, Lian Chen, Yi Lu, Xin-Bao Xie, Kuerbanjiang Abuduxikuer, Jian-She Wang

**Affiliations:** 1 Center for Pediatric Liver Diseases, Children’s Hospital of Fudan University, Shanghai, China; 2 Department of Pediatrics, Icahn School of Medicine at Mount Sinai, New York, NY USA; 3 Department of Pathology, Children’s Hospital of Fudan University, Shanghai, China

**Keywords:** Neonatal cholestasis, Acid sphingomyelinase deficiency, High-density lipoprotein cholesterol

## Abstract

**Background:**

Patients with acid sphingomyelinase deficiency (ASMD) may be referred to a hepatologist for liver manifestations. This study summarized the liver manifestations of patients with ASMD in the early disease course.

**Methods:**

This study enrolled ASMD patients diagnosed by genetic tests between July 2016 and December 2020 in a national pediatric liver center. The significance of low High-density lipoprotein cholesterol (HDL-C) for aid diagnosis of ASMD in infancy was explored by reviewing 160 consecutive infants with liver manifestations, who underwent both genetic tests and lipid profile studies, between January 2020 and December 2020.

**Results:**

A total of 7 patients were diagnosed as ASMD, and 10 known disease-causing variants were identified. Hepatosplenomegaly, elevated transaminases, and liver foam cells were observed in all the 7 patients at age ranging from 4 to 31 months. Low HDL-C was detected in 5 patients, cherry red spot in 4 patients, development delay in 3 patients, and interstitial lung diseases in 1 patient. Three ASMD patients developed cholestasis around 1 month of age, and bilirubin levels normalized at age ranging from 3 to 10 months. They had persistently elevated transaminases and hepatosplenomegaly, and died within 4 years of age. Among the 160 infants with liver manifestations, 125 (78.1%) had low HDL-C. Fifty-four had both low HDL-C and splenomegaly, including 48 cholestatic infants, but only 1 (1.9%, 1/54) infant without cholestasis was diagnosed as ASMD.

**Conclusions:**

ASMD can manifest as neonatal cholestasis in the early disease course. Cholestasis is a pitfall when low HDL-C is used for aid diagnosis of ASMD in infants with splenomegaly.

## Background

Acid sphingomyelinase deficiency (ASMD), a rare and progressive lysosomal storage disorder, results from biallelic pathogenic variants in *SMPD1* [1, 2]. *SMPD1* encodes acid sphingomyelinase, which catalyzes hydrolysis of sphingomyelin to ceramide and phosphocholine. ASMD can lead to progressive accumulation of sphingomyelin and other lipids in mononuclear phagocytic system, and manifests as a multi-system disease involving liver, spleen, bone, lung, and nervous system. ASMD is traditionally categorized as infantile neurovisceral ASMD (also known as Niemann-Pick disease type A, NP-A), chronic visceral ASMD (also known as NP-B), or chronic neurovisceral ASMD (the intermediate form) [2]. The estimated incidence is about 1:250,000 live birth [3].

A diagnosis of ASMD is established if biallelic pathogenic variants were identified in *SMPD1* and / or activity of ASM enzyme decreased [2, 4, 5]. Common presentations for ASMD patients are elevated transaminases, low high-density lipoprotein cholesterol (HDL-C), hepatosplenomegaly, developmental delay, hypotonia, cherry red maculae, and interstitial lung disease [2]. Although care of ASMD patients is primarily provided by metabolic specialists, ASMD patients may also be referred to a hepatologist for liver manifestations in the early disease course, even for jaundice [6–8]. However, knowledge on the early liver manifestations of ASMD patients is deficient. Detailed descriptions of them may help to early identify patients with a high clinical suspicion of ASMD. After common causes of liver manifestations are excluded, genetic tests are routinely ordered in our center if inherited diseases are suspected [9]. It provides a chance to detail the early liver manifestations of ASMD patients.

In this study, we report the clinical findings of 7 pediatric ASMD patients confirmed by genetic tests in the early disease course, and unexpectedly found that 3 of them presented as neonatal cholestasis.

## Methods

**Fig. 1:**
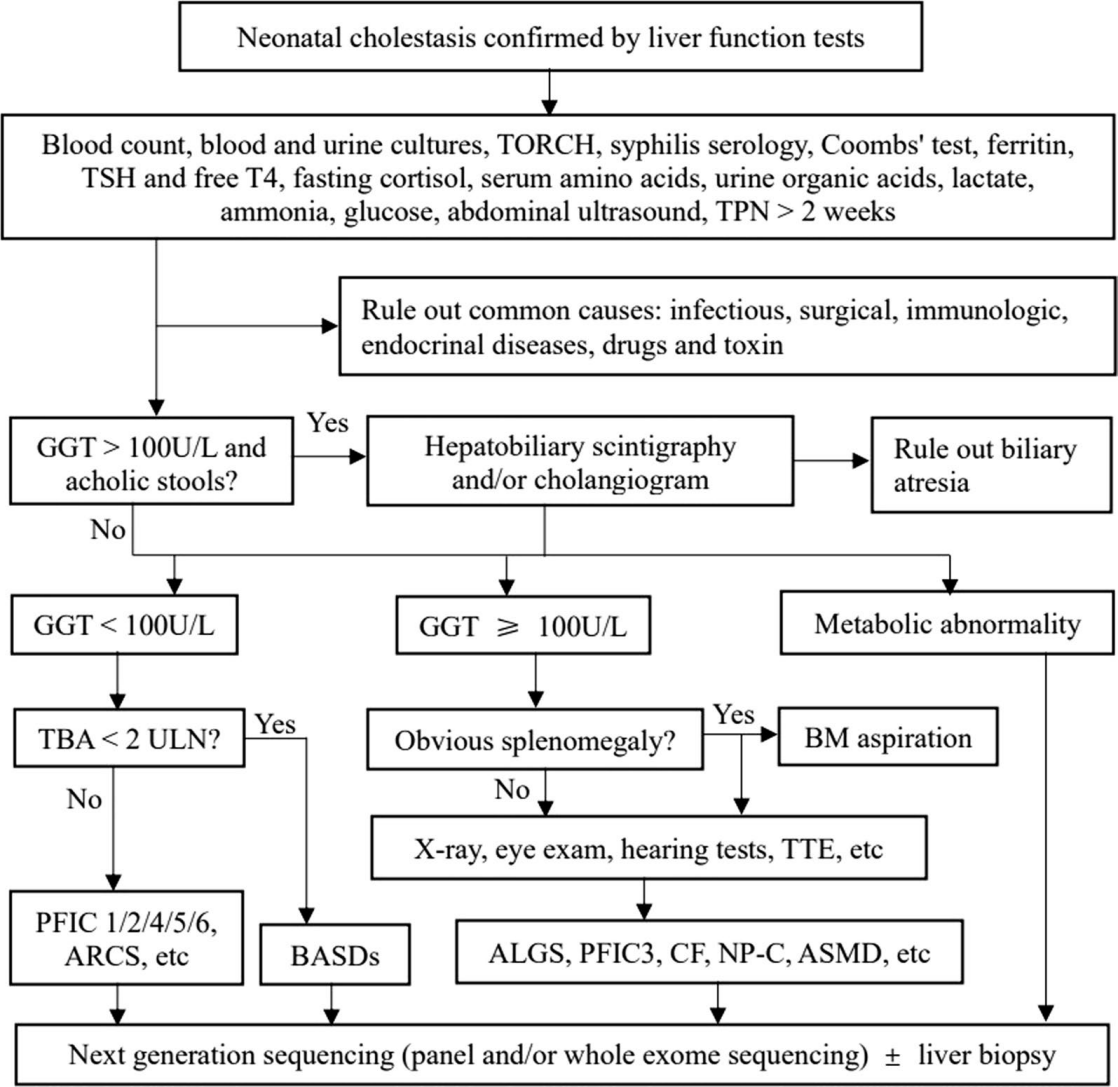
A diagnostic algorithm for neonatal cholestasis. TPN, total parenteral nutrition; GGT, γ-glutamyl transpeptidase; TBA, total bile acids; ULN, upper limit of normal value; BASD, bile acid synthesis defect; PFIC, progressive familial intrahepatic cholestasis; ARCS, arthrogryposis, renal dysfunction, and cholestasis syndrome; TTE, transthoracic echocardiography; BM, bone marrow; ALGS, alagille syndrome; CF, cystic fibrosis; NP-C, Niemann-Pick disease type C; ASMD, acid sphingomyelinase deficiency

Between July 2016 and December 2020, 1169 inpatients, who were referred to the Center for Pediatric Liver Diseases, Children’s Hospital of Fudan University for subspecialty investigations of liver manifestations, received genetic tests, including panel (n = 555), medical exome (n = 100), and/or whole exome sequencing (n = 514). The patients aged from 1 month to 17 years old. Before genetic analyses were ordered, common causes of liver manifestations, including infectious, surgical, immunologic, endocrinal diseases, drugs and toxin, etc. were excluded. A diagnostic algorithm for neonatal cholestasis was presented in Fig. 1. Genetic tests were performed in the Translational Center of Children’s Hospital of Fudan University, data analyses and variation classification were done as described previously [10, 11]. NM_000543 was used as *SMPD1* reference sequence. Variant pathogenicity was assessed following the American College of Medical Genetics and Genomics (ACMG) standards and guidelines [12]. The diagnosis of ASMD was established if biallelic pathogenic or likely pathogenic variants were identified.

Histologic studies were done at the Department of Pathology of the same hospital. Liver tissues sections were stained by hematoxylin & eosin (HE), periodic acid-schiff (PAS), masson staining, etc. Bone marrow aspirations were Wright’s stained. Liver inflammation activity and fibrosis grade were assessed according to the Scheuer scoring system [13].

To explore the significance of low HDL-C for aid diagnosis of ASMD in infancy, this study enrolled 160 consecutive infants with liver manifestations, who were referred to the same center and underwent both genetic tests and lipid profile studies, between January 2020 and December 2020.

Clinical data were retrospectively gathered from the medical records. Hepatomegaly and splenomegaly were diagnosed by ultrasonography. Cholestasis was defined as direct bilirubin (DB) level > 17.1 μmol/L when the total bilirubin (TB) is < 85.5 μmol/L or the DB is > 20.0% of the TB if the TB is > 85.5 μmol/L [14]. Cholestasis onset within 3 months of age was defined as neonatal cholestasis. Pathogenic variants in other cholestasis causing genes were also analyzed if the ASMD patients presented as neonatal cholestasis.

This study was conducted according to the Declaration of Helsinki and approved by the ethics committees of the Children’s Hospital of Fudan University (2021–17). Informed consent had been obtained from the parents/guardian during the admission.

### Statistical analysis

Comparison of two medians was done by nonparametric Mann–Whitney test using the SPSS Inc. version 17.0 software (University of Chicago, Chicago). Difference among ratios was tested by Chi-square test using Fisher’s exact value. *P* < 0.05 was considered significant.

## Results

### Molecular findings

**Fig. 2:**
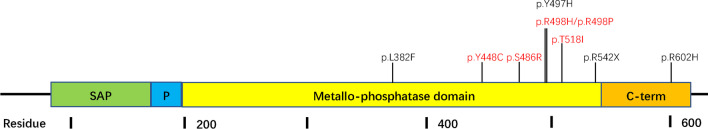
Distribution of *SMPD1* variants in the ASM domains. *SMPD1* variants identified in ASMD patient with neonatal cholestasis are shown in red

A total of 7 patients were diagnosed as ASMD for harboring biallelic or possible biallelic *SMPD1* pathogenic variants (Table 1). Ten distinct variants were identified in *SMPD1*, including 1 intronic, 1 nonsense, and 8 missense variants. One common missense variant, c.1458 T > G (p.S486R), was identified in 4 of the 7 ASMD patients. All the 10 *SMPD1* variants were known disease-causing variants for ASMD [5, 6]. The nonsense variant and 8 missense variants located in the last 4 exons of SMPD1 gene (exon 3–6), which encoding a catalytic metallophosphatase domain and a helical C-termina domain (Fig. 2). The intronic variant located at flanking intronic region of exon 5 of SMPD1 gene. Parental studies were performed in patient (P) 2, P5, and P6, revealing that all were compound heterozygous.

**Table 1:** General information, molecular and clinical findings of 7 ASMD patients

	Gender	First symptoms / Age at first symptoms	*SMPD1* variants (NM_000543)	First available liver biochemistry results						Outcome
				Age	TB	DB	ALT	AST	GGT	
P1	Female	Failure to thrive / 6 months	c.1486 + 5G > C / c.1144C > T (p.L382F)	27 months	5.4	0.4	88	115	18	Lost to follow-up
P2	Male	Abdominal distention / 11 months	c.1458 T > G (p.S486R) / c.1805G > A (p.R602H)	15 months	17.3	6.3	297	412	61	HCST at 4 years old. Alive, 5 years and 3 months
P3	Female	Jaundice / 1 month	c.1458 T > G (p.S486R) / c.1343C > T (p.Y448C)	5 months	47.5	25.3	113	203	128	Died, 3 years and 1 months
P4	Male	Jaundice / 1 month	c.1458 T > G (p.S486R) / c.1553C > T (p.T518I)	4 months	168.0	114.5	19	221	197	Died, 2 years and 6 months
P5	Male	Abdominal pain / 26 months	c.1489 T > C (p.Y497H) / c.1489 T > C (p.Y497H)	26 months	15.3	9.9	105	97	33	Alive, 4 years and 11 months
P6	Male	Failure to thrive / 6 months	c.1624C > T (p.R542X) / c.1458 T > G (p.S486R)	6 months	7.7	2.7	246	346	na	Alive, 1 year and 9 months
P7	Male	Jaundice / 1 month	c.1493G > A (p.R498H) / c.1493G > C (p.R498P)	1.5 months	83.5	36.7	146	438	356	Died, 3 years and 10 months

P, patient; ASMD, acid sphingomyelinase deficiency; TB (μmol/L), total bilirubin; DB (μmol/L), direct bilirubin; ALT (U/L), alanine aminotransferase; AST (U/L), aspartate aminotransferase; GGT (U/L), γ-glutamyl transpeptidase; na, not available; HCST, hematopoietic stem cell transplantation

Three patients (P3, P4, and P7) presented as neonatal cholestasis (Table 1). Apart from a *CFTR* (NM_000492) heterozygous known pathogenic variant, c.3209G > A (p.R1070Q), was identified in P4, no additional pathogenic variant was identified in other cholestasis causing genes.

### Clinical presentations

The 7 ASMD patients came from 7 distinct healthy non-consanguineous families after uneventful pregnancies. All were born at term, with birth weight ranging from 3050 to 3500 g. Three patients (P3, P4, and P7) developed jaundice around 1 month of age. They were first investigated for prolonged jaundice at age of 5 months, 4 months, and 1.5 months respectively. Cholestasis with high γ-glutamyl transpeptidase (GGT > 100 U/L) was confirmed by liver function tests (Table 2). Two patients (P1 and P6) were first investigated for failure to thrive, and elevated transaminases were found in both patients at age of 27 months and 6 months respectively. P2 and P5 were found to have elevated transaminases and hepatosplenomegaly for investigation of abdominal distention or pain at age of 15 months and 26 months respectively.

**Table 2:** Further evaluation of ASMD patients at referral

	P1	P2	P3	P4	P5	P6	P7
Age (months)	31	16	8	4	26	7	6
Evolution							
Hepatomegaly	+	+	+	+	+	+	+
Splenomegaly	+	+	+	+	+	+	+
Developmental delay	+	+	−	−	−	+	−
Cherry red spot	ND	ND	+	+	−	+	+
Hypotonia	−	−	−	−	−	−	−
Interstitial lung disease	+	−	−	−	−	−	−
Biochemistry results							
Total bilirubin (μmol/L)	7.3	20.3	33.3	100.4	13.7	8.7	10.9
Direct bilirubin (μmol/L)	1.4	5.3	20.2	57.9	6.6	3.4	2.9
Alanine aminotransferase (U/L)	31	63	118	20	121	239	54
Aspartate aminotransferase (U/L)	57	103	368	214	126	230	210
γ-glutamyl transpeptidase (U/L)	16	38	195	167	38	66	37
Total bile acid (μmol/L)	7.8	2.4	92.6	329.5	6.9	10.9	27.2
HDL-C (mmol/L)	ND	0.42	0.71	ND	0.67	0.74	0.72
Platelet (× 10^9/L)	90	204	103	327	140	329	189
Foam cells in bone marrow	+	+	+	+	+	+	+
Liver histologic studies							
Foam cells	+	+	+	+	+	+	+
Cholestasis	−	−	−	+	−	−	−
Activity grade	G0	G0	G0	G1	G0	G1	G0
Fibrosis stage	S1	S3	S3	S2	S2	S2	S1

ND, not done; HDL-C (mmol/L), high density liptein cholesterol (ref: 1.03–1.55)

**Fig. 3:**
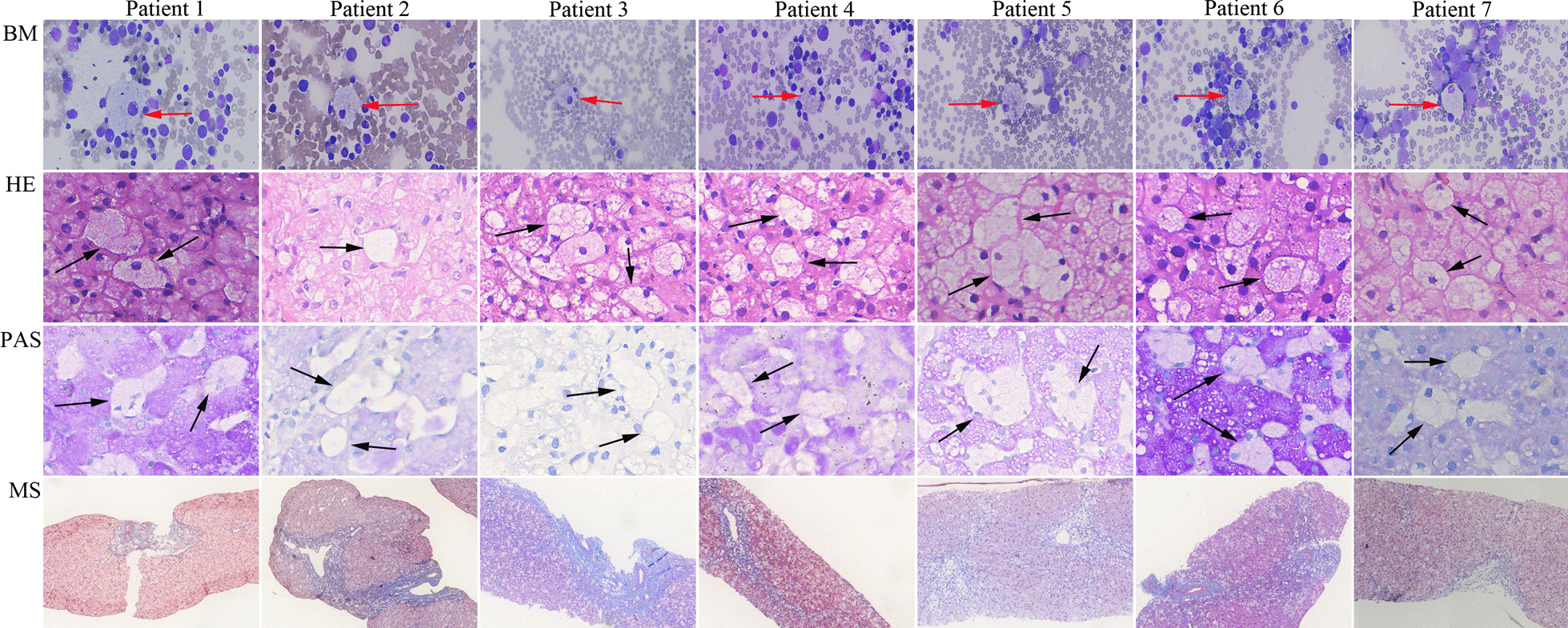
Histologic studies of bone marrow aspirations and liver tissues obtained from ASMD patients. BM (400x), bone marrow; HE (400x), hematoxylin & eosin; PAS (400x), periodic acid-schiff; MS, masson (40x). Foam cells are detected in both bone marrow (red arrow) and liver tissues (black arrow)

When the 7 ASMD patients were referred to our hospital for subspecialty evaluations, all had hepatosplenomegaly and elevated aspartate aminotransferase (AST) with the ratios of AST to alanine aminotransferase (ALT) ranging from 0.9 to 10.7 (Table 2). Lipid profile studies were monitored in 5 patients (P2, P3, and P5 ~ P7), and revealed low HDL-C in all 5 patients. Cherry red spot was identified in 4 patients (P3, P4, P6, and P7). Developmental delay was observed in 3 patients (P1, P2, and P6). Lung CT scan was performed in P1 and revealed interstitial lung disease (ILD), while chest X-ray was done in P2-P7 and did not show ILD. Hypotonia was not recorded in all 7 patients. Bone marrow aspiration and liver biopsy were performed in all 7 ASMD patients at ages ranging from 4 to 31 months. Foam cells were detected in all bone marrow samples and liver tissues (Fig. 3).

### The evolution of ASMD patients presenting as neonatal cholestasis

P3, P4 and P7 were referred to us at the ages of 8, 4 and 6 months respectively. At referring, direct bilirubin was still elevated in P3 (20.2 μmol/L) and P4 (57.9 μmol/L), but spontaneously normalized in P7 (2.9 μmol/L) (Table 2). Apart from abnormal international normalized ratio (INR = 1.86) that was responsible to vitamin K1 administration in P4, no abnormality was found on INR or the levels of 25-hydroxy vitamin D3 (25-OH-D3) in other patients. Ursodesoxycholic acid (UDCA) and fat-soluble vitamins were given to P3 and P4, bilirubin level was normalized in them at age of 10 months and 9 months respectively. However, all 3 patients had persistently elevated transaminases and hepatosplenomegaly after jaundice disappeared, and all died within 4 years old (Table 1).

### Low HDL-C in infants with liver manifestations

Of the 160 enrolled infants with liver manifestations, 111 were referred for cholestatic jaundice and 49 for other liver presentations. Low HDL-C was detected in 125 (78.1%) of the 160 infants. Among infants with cholestatic jaundice, HDL-C levels were similar between patients within and those beyond 6 months of age; the same was found among patients with other liver presentations (all *P* > 0.05) (Table 3). Among infants within 6 months of age, HDL-C levels in infants with cholestatic jaundice were lower than that in infants with other liver presentations; the same was found among patients beyond 6 months of age (all *P* < 0.05).

**Table 3:** Low HDL-C in infants with liver manifestations

	Infants with cholestatic jaundice			Infants with other liver presentations		
	Total (n = 111)	Within 6 months of age (n = 97)	Beyond 6 months of age (n = 14)	Total (n = 49)	Within 6 months of age (n = 19)	Beyond 6 months of age (n = 30)
Sex (male/female)	74/37	64/33	10/4	36/13	16/3	20/10
Age (months)	3 [2, 4]	2.0 [2.0, 4.0]	8.5 [7.0, 9.0]	6 [4, 8]^*^	3.0 [3.0, 4.0]	8.0 [6.0, 9.0]
HDL-C levels (mmol/L)^#^	0.70 [0.56, 0.85]	0.70 [0.59, 0.86]	0.56 [0.49, 0.75]	1.02 [0.82, 1.22]^*^	0.96 [0.82, 1.18]^*^	1.03 [0.90, 1.23]^*^
Low HDL-C (%)	100 (90.1%)	88 (90.7%)	12 (85.7%)	25 (51.0%)^*^	11 (57.9%)^*^	14 (46.7)^*^
With splenomegaly	48	44	4	6	3	3
Without splenomegaly	52	44	8	19	8	11
ASMD	0	0	0	1	0	1

Interquartile range in square brackets

HDL-C, high-density lipoprotein cholesterol; ASMD, acid sphingomyelinase deficiency

^#^Normal reference: 1.03–1.55 mmol/L

*Infants with cholestatic jaundice vs. infants with other liver presentation, *P* < 0.05

A total of 54 infants, including 48 with cholestasis, had both low HDL-C (ref: 1.03–1.55 mmol/L) and splenomegaly. Among them, 1 infant (P6) without cholestasis was finally diagnosed as ASMD by genetic tests. The ratio of positive diagnosis of ASMD was 1.9% (1/54).

## Discussion

ASMD patients are usually diagnosed at a median age of 5.37 years, with only a few cases are diagnosed within the first year of life [5, 6]. Although jaundice has been reported in a few ASMD infants diagnosed within the first year of life [6–8], hepatosplenomegaly and elevated transaminases are common reasons why ASMD patients are referred to a hepatologist. In this study, 7 patients with liver manifestations were diagnosed as ASMD by genetic tests in early disease course, including 3 presenting as neonatal cholestasis. To our best knowledge, this is the first report to provide evidence that ASMD can present as neonatal cholestasis.

Both ASMD and NP-C are subtypes of Niemann-Pick disease resulting from lysosomal accumulations [15]. NP-C is a known cause of neonatal cholestasis [16, 17]. It is believed that infantile neurovisceral ASMD patients can also manifest cholestatic jaundice [2]. In current cases series, 3 ASMD patients presented as neonatal cholestasis, in whom other known causes of neonatal cholestasis were excluded following a comprehensive work-up [9, 18]. We also excluded a possibility that neonatal cholestasis of these 3 ASMD patients resulted from defects of other neonatal cholestasis-causing genes. Their cholestasis resolved spontaneously or after UDCA administration at age ranging from 3 to 10 months, while hepatomegaly, splenomegaly and elevated transaminases persisted. These are closely similar to NP-C [19, 20]. Therefore, it is reasonable to conclude that neonatal cholestasis can be an early liver manifestation of ASMD patients.

The presence of following suggestive features, such as developmental delay, cherry red maculae, hypotonia, and low HDL-C, raises a suspicion of ASMD in infants and children with splenomegaly [2]. In this study, low HDL-C was indeed detected in all 5 patients who had lipid profile tested. It has been hypothesized that low HDL-C may be attribute to the accumulation of sphingomyelin within the liver [21]. However, low HDL-C is also commonly detected in infants with liver manifestations, especially in those with cholestasis. Therefore, cholestasis may be a pitfall when low HDL-C is used for aid diagnosis of ASMD in infants with splenomegaly. Liver foam cells were detected in all 7 ASMD patients at age ranging from 4 to 31 months, and indicative of ASMD.

The 3 ASMD patients presenting as neonatal cholestasis with splenomegaly also had high GGT. The diagnosis may be missed, because ASMD is not listed as a differential diagnosis of neonatal cholestasis, and neonatal cholestasis with high GGT commonly results from biliary atresia (BA), neonatal intrahepatic cholestasis caused by citrin deficiency (NICCD), ALGS, progressive family intrahepatic cholestasis type 3 (PFIC3), cystic fibrosis, etc. [16, 17]. However, splenomegaly is not a common feature of these disorders mentioned above in the early disease course, and neonatal cholestasis with splenomegaly usually raises a clinical suspicion of NP-C [22]. For avoiding misdiagnosis, we suggest that ASMD can be listed as a differential diagnosis of neonatal cholestasis with both high GGT and splenomegaly. Cherry red maculae is detected in all the 3 ASMD patients, and raises a high suspicion of ASMD in patients with splenomegaly. However, it develops with age, and can usually be negative in the early disease course [4]. In conditions without cherry red maculae, liver foam cells can raise a clinical suspicion of ASMD, but also NP-C [23]. Genetic tests are needed to distinguish ASMD from NP-C, and can lead to a definite diagnosis.

In summary, we herein report the molecular findings and liver manifestations in early disease course of 7 children with ASMD. Three present as neonatal cholestasis with high GGT. ASMD should be listed as a differential diagnosis of neonatal cholestasis with splenomegaly and high GGT. Cholestasis is a pitfall when low HDL-C is used for aid diagnosis of ASMD in infants with splenomegaly.

## Data Availability

The datasets used and/or analyzed during the current study available from the corresponding author on reasonable request.

## Abbreviations

ASMD: Acid sphingomyelinase deficiency
HDL-C: High-density lipoprotein cholesterol
ACMG: American College of Medical Genetics and Genomics
NP-C: Niemann-Pick disease type A
UDCA: Ursodesoxycholic acid
INR: International normalized ratio
ILD: Interstitial lung disease
GGT: γ-Glutamyl transpeptidase
AST: Aspartate aminotransferase
ALT: Alanine aminotransferase
DB: Direct bilirubin
TB: Total bilirubin
